# Calisthenics

**Published:** 1890-05

**Authors:** Melvin L. Severy


### CALISTHENICS.

BY MELVIN L. SEVERY. (IN THE ESOTERIC.)

The word "calisthenics" is derived from two Greek words, and signifies "beautiful strength."

The system of physical culture practised by the ancient Greeks may well be considered as one of the lost arts ; for nowhere is it now in use ; and throughout this country to-day there are not a dozen institutions where any system of physical development worthy of the name of calisthenics is practised. The effect of this deplorable fact is only too plainly seen by a visit to our schools and colleges, where that prologue to consumption, the "scholar's stoop," is seen on every side. Perhaps not ten in thirty wholly escape it, and those ten will generally be found to possess an ungainly and ill-balanced strength, in many cases even more destructive than the "scholar's stoop" itself ; for if there is any one thing which is productive of disease more than another, that thing is inharmony.

The "scholar's stoop" consists of the following deformities. Hollow chest, projecting scapulæ or shoulder-blades, and generally an abnormal break in the spine near the small of the back, causing a constitutional weakness of the waist muscles. Its remedy is not the gymnasium—for it is not uncommon there—but a system of calisthenics based upon the best physiological data. Such a system will be noticed further on.

A word about our gymnasiums, and, let it be understood, I refer to them only when acting at their best, neglecting to detail the various injuries from falls, sprains, ruptures, etc. The tendency of the modern gymnasium is to pile up muscle on the extremities of the body at the expense of the vital organs. Now, inasmuch as it is a physiological law that an overdeveloped muscle will draw its strength from a weaker one, lack of harmony becomes dangerous. Suppose, however, that the *danger* be not recognized, what is the *use* of making the arms half again as strong as some other member of the body? As the strength of a chain is that of its weakest link, so the strength of the body is that of its weakest part.

The serious effect of inharmonious development is shown in the following instance.

A young student joined a Boston gymnasium, taking the dumb-bell, cross-bar, ladder, and various other exercises for his health. The result was that his arms became developed out of all harmony with the rest of his body, and now he repeatedly strains himself by lifting weights which his waist muscles are incapable of sustaining. It will readily be seen that, if the law of harmonious development had been faithfully observed, one member would be *incapable* of lifting a weight sufficient to strain another member.

The power and activity of the muscles about the vital organs should, in maintenance of the laws of harmony and of division of labor, greatly exceed that of any other portion of the body; otherwise indigestion, dyspepsia, and other kindred complaints are certain to exist with some degree of violence. The gymnasium of to-day works in direct opposition to the above principle.

Now let us suppose a man goes to the gymnasium and succeeds in obtaining all the muscle desirable, is he physically cultured? By no means. In nine cases out of ten he will be stiff and awkward, and, if asked to make a *gesture*, will simply execute such a *movement* as will bespeak him to be all of one piece.

What, then, is needed? A system of calisthenics which shall embody the following salient points.

1. A development of all the muscles in their proper proportions, the predominance being given to those about the vital organs.

2. A FREEING (as well as development) of all the muscles, which will produce not only *powerful*, but *beautiful* movement.

3. Exercises which shall act directly upon the nerve force to strengthen and balance it.

4. The practice of healthful respiration, a thing almost entirely neglected in our gymnasiums, and that, too, with most unpleasant results.

5. The possibility of practising said system at any time or place without the *expense* of any apparatus or *teacher*; thus placing healthful exercise within the reach of all.

The system of calisthenics to which I refer is the result of an extended and minute study of anatomy in the dissecting room. It contains, among others, the following exercises, every one of which draws vigorously upon the vital organs.

| | |
|---|---|
| Reaching............. | Exercises. |
| Waist | " |
| Bending | " |
| Poising | " |
| Breathing | " |
| Voice | " |

As well as exercises of the chest, neck, etc.

In the performance of each one of the above exercises the laws of beauty, unity, continuity of line, opposition, succession, economy, etc., are implicitly obeyed.

I will describe a few of the most important exercises, giving attention to those which will be of the most service to the reader from the standpoint of *health*.

### THE WAIST EXERCISE.

If a portion of the body be pinched severely, it will, after relaxation, become red from the blood which rushes to the spot. This is a physiological law throughout the body; and the blood will not properly circulate through the stomach and liver *without* this alternate pressure and relaxation; and, moreover, if the blood does *not* so circulate, the liver will not efficiently secrete bile, nor the stomach, gastric juice; hence indigestion is sure to ensue. In other words, for health, the stomach and liver must be literally *churned* with every breath from *birth to death*. The aim of the waist exercise is to bring about this healthful state of affairs—a task it readily accomplishes—and I may add here that this exercise properly and persistently employed, is a certain, absolute, and permanent cure for the most stubborn case of dyspepsia. I have now in mind a clergyman who was cured, by this system of calisthenics, of dyspepsia of fifteen years standing.

There are two distinct standing positions, namely: the venous position, or, with the weight on the heels; and the arterial position, or with the weight on the balls of the feet.

All of these exercises should be taken from the arterial standing position, and it may be further added, that many persons who suffer from pain in the back, during protracted standing, will find in the arterial standing position an *instantaneous* and *effectual* remedy.

### DIRECTIONS FOR WAIST EXERCISE.

Take arterial position with feet at an angle of forty-five degrees; knees straight. Place the palms of the hands upon the hips, thumbs forward, fingers down. Bend the body at a point as near the lower end of the ensiform cartilage as possible, griping the stomach as in a *vise.* Press the body down upon the stomach *until quite painful.* Do *not* bend the hips. Now turn the torso around upon this pivot formed near the pit of the stomach, describing as large a circle as possible with the shoulders. Keep the entire circle regular, a difficulty which will be especially felt when passing over each hip. N. B. In the vicinity of the waist there are two natural joints; one at the hips, and the other above. The hip joint must *not* be used in this exercise. It is that great, universal joint at the very *belt of life*, generally ignored, and with most serious results, that this exercise is calculated to free, strengthen and develop.

No "lady of fashion" ever uses this joint; for, from early womanhood, she has taken the utmost precaution to make this great pivot of life as inflexible as the steel which "*supports*" it; and yet will she complain of dyspepsia—the very disease she has been at such pains to induce.

The poising exercises are especially calculated to act directly upon, and strengthen, the nerves. I have known of severe cases of nervous debility (notably one where a shock had already occurred) being cured by a careful and persistent practice of poising.

